# Distinguishing Molecular Features and Clinical Characteristics of a Putative New Rhinovirus Species, *Human Rhinovirus C* (HRV C)

**DOI:** 10.1371/journal.pone.0001847

**Published:** 2008-04-02

**Authors:** Peter McErlean, Laura A. Shackelton, Emily Andrews, Dale R. Webster, Stephen B. Lambert, Michael D. Nissen, Theo P. Sloots, Ian M. Mackay

**Affiliations:** 1 Queensland Paediatric Infectious Diseases Laboratory, Sir Albert Sakzewski Virus Research Centre, Royal Children's Hospital, Brisbane, Queensland, Australia; 2 Clinical and Medical Virology Centre, University of Queensland, Brisbane, Queensland, Australia; 3 Mueller Laboratory, Center for Infectious Disease Dynamics, Department of Biology, The Pennsylvania State University, University Park, Pennsylvania, United States of America; 4 Department of Biochemistry and Biophysics, University of California San Francisco, San Francisco, California, United States of America; 5 Biological and Medical Informatics Program, University of California San Francisco, San Francisco, California, United States of America; 6 Howard Hughes Medical Institute, University of California, San Francisco, California, United States of America; 7 Division of Microbiology, Queensland Health Pathology Service, Royal Brisbane Hospitals Campus, Brisbane, Queensland, Australia; 8 Department of Paediatrics and Child Health, Royal Children's Hospitals, Brisbane, Queensland, Australia; University of Hong Kong, China

## Abstract

**Background:**

Human rhinoviruses (HRVs) are the most frequently detected pathogens in acute respiratory tract infections (ARTIs) and yet little is known about the prevalence, recurrence, structure and clinical impact of individual members. During 2007, the complete coding sequences of six previously unknown and highly divergent HRV strains were reported. To catalogue the molecular and clinical features distinguishing the divergent HRV strains, we undertook, for the first time, *in sili*co analyses of all available polyprotein sequences and performed retrospective reviews of the medical records of cases in which variants of the prototype strain, HRV-QPM, had been detected.

**Methodology/Principle Findings:**

Genomic analyses revealed that the six divergent strains, residing within a clade we previously called HRV A2, had the shortest polyprotein of all picornaviruses investigated. Structure-based amino acid alignments identified conserved motifs shared among members of the genus *Rhinovirus* as well as substantive deletions and insertions unique to the divergent strains. Deletions mostly affected regions encoding proteins traditionally involved in antigenicity and serving as HRV and HEV receptor footprints. Because the HRV A2 strains cannot yet be cultured, we created homology models of predicted HRV-QPM structural proteins. *In silico* comparisons confirmed that HRV-QPM was most closely related to the major group HRVs. HRV-QPM was most frequently detected in infants with expiratory wheezing or persistent cough who had been admitted to hospital and required supplemental oxygen. It was the only virus detected in 65% of positive individuals. These observations contributed to an objective clinical impact ranging from mild to severe.

**Conclusions:**

The divergent strains did not meet classification requirements for any existing species of the genus *Rhinovirus* or *Enterovirus*. HRV A2 strains should be partitioned into at least one new species, putatively called *Human rhinovirus C*, populated by members detected with high frequency, from individuals with respiratory symptoms requiring hospital admission.

## Introduction

Human rhinoviruses (HRVs) are the most frequently detected pathogens in acute respiratory tract infections (ARTIs). HRVs have been associated with lower respiratory tract (LRT) illness and more serious clinical outcomes within pediatric and other vulnerable populations [1]. Despite this, HRV strains continue to be commonly defined, *en masse*, by their most prolific and currently most well-defined role in causing the ‘common cold’ [2], [3].

Classified within the family *Picornaviridae*, the genus *Rhinovirus* consists of 100 serotyped strains divided into two species, *Human rhinovirus A* (HRV A; n = 75) and *Human rhinovirus B* (HRV B; n = 25) [4]. Species classification was initially based on the susceptibility of strains to capsid binding antivirals [5], [6] and subsequently confirmed by phylogenetic studies [4], [7]. HRVs are also subdivided by receptor usage; major group HRVs use an intercellular adhesion molecule (ICAM-1; n = 88 strains), which interacts within a depression of the viral surface known as the canyon [8]. Minor group HRVs (n = 12) receive a molecule from the low density lipoprotein receptor family (LDLR) at protrusions along the 5-fold axis of the capsid surface [9]–[12]. Recently, heparan sulphate was identified as a pH dependent, low efficiency receptor for HRV-54 [13]. A relationship between receptor usage and species identity is only evident among minor group HRVs; all are HRV As. Major group HRVs reside in both species. Studies have identified four neutralizing immunogenic sites on the surface of a major group HRV (HRV-14; NImIA, NImIB, NImII and NImIII)[14] and three sites on a minor group HRV (HRV-2; site A, B and C) [15], with some overlap [16]. These sites consist of discontinuous amino acid sequence within VP1, VP2 and VP3.

The human enteroviruses (HEVs) are the closest genetic relatives of the HRVs but they belong to the genus *Enterovirus*. HEV strains are classified into four species (A–D) and some have been implicated in ARTI [17]. Similar to the major group HRVs, some HEVs have been shown to use ICAM-1 as their primary cellular receptor [18]. Other host molecules employed by HEVs include the decay accelerating factor (DAF) [19], [20], poliovirus receptor (PVR) [21] and coxsackie-adenovirus receptor (CAR) [22]. Under a proposal currently before the International Committee on Taxonomy of Viruses (ICTV), HRV and HEV strains will be combined into the genus *Enterovirus*, at the expense of *Rhinovirus*, an issue of contention for decades [23].

**Figure 1:**
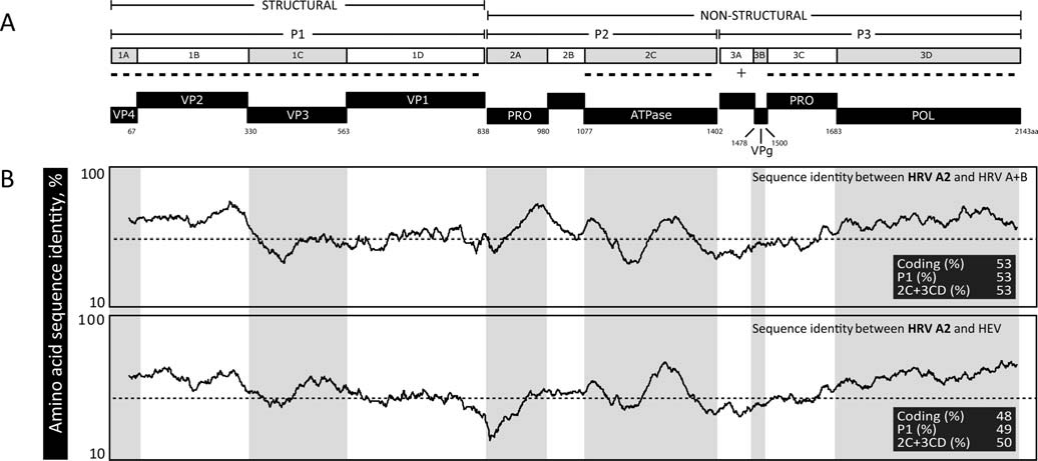
Characterization of HRV A2 genomes. (A) Scale representation of the coding region from prototype HRV A2 virus, HRV-QPM. Predicted protein products from the structural (VP4 to VP1) and non-structural (PRO-protease; POL-polymerase) regions are indicated. Regions are shaded alternatively for convenience and numbering indicates positions of predicted protease cleavage according to the start of the HRV-QPM polyprotein. Dashed lines indicate regions involved in species classifications. (B) HRV A2 amino acid similarity to HRV A (n = 34) and B (n = 13), or HEV (n = 71) strains as determined by SimPlot© analysis. Plots indicate the percentage similarity of a 50% consensus sequence from each species' polyprotein compared to the HRV A2 strains. Average similarity across the polyprotein is indicated by dotted line. Averaged values for the complete coding, P1 and 2C+3CD regions are indicated within the inset of each panel.

Picornavirus genomes typically consist of a 7.2 to 7.5 kb positive-sense, single-stranded RNA molecule located within a non-enveloped icosahedral capsid. The genomes contain a single open reading frame flanked by 5′and 3′untranslated regions (UTR). The polyprotein is first cleaved into three precursor polyproteins, P1, P2 and P3 (Figure 1). P1 is further cleaved into the structural proteins VP4, VP2, VP3 and VP1. Union of a single copy of each structural protein constitutes a viral protomer; the HRV capsid consists of 60 protomers arranged in a T = 1, pseudo T = 3 (i.e. T = p3) conformation. P2 and P3 are cleaved into seven non-structural proteins including two proteases (2A^pro^ and 3C^pro^) and an RNA-dependant RNA polymerase (3D^pol^) [24].

Recent studies have generated 27 additional HRV A and 12 additional HRV B genomes [25], [26] also resulting in the proposal that individual HRV genomes are under purifying selective pressure and that significant intra-strain variation occurs only at the antigenic sites [26]. This is in stark contrast to the HEVs for which recombination in the non-structural regions is a significant contributor to viral diversification [27], [28].

Untypeable rhinoviruses have been described using PCR-based tools, since 1994 [29] but it was only in 2007 that the complete coding sequences of a number of novel and apparently divergent HRV strains were reported, permitting a for identification of the first new strains in two decades [30]. The initial strain described, HRV-QPM, was identified from children with presenting symptoms often suggesting bronchiolitis [31]. Subsequently, ‘HRV-Xs’ were found in an adult asthma study in the United States [32] and ‘HRV-Cs’ were described from a study of pediatric infection by human bocavirus in Hong Kong [33]. Some notable features associated with these six putative viruses include the reliance upon molecular methods for their detection, their apparent endeminicity, their failure to produce cytopathic effects in culture, their frequent detection in subjects with expiratory wheezing and their limited sequence identity with existing HRV strains but high shared sequence identity. Studies employing subgenomic sequences proposed that there were many of these putative viruses occupying a distinct position within the genus *Rhinovirus*, which we collectively called HRV A2 [34]. It has been proposed that these strains may constitute a new picornavirus species [33]. Data have not yet been presented which appropriately address classification using current ICTV criteria and the scope of the clinical impact of these divergent strains has not yet been studied in detail. Because it appears that the members of this clade are frequently associated with LRT illness we sought to better characterise them in the hope of finding features that might prove useful in predicting the outcome of HRV A2 infection.

Intensive genomic analysis and computer-based modelling resulted in comprehensive characterisation of all HRV A2 polyprotein sequences and, by superimposing the sites of known receptor contacts, we were able to propose a more robust taxonomic placement for these newly identified viruses (NIVs). Detailed medical chart reviews on HRV-QPM-positive (prototype HRV A2) individuals were augmented by a severity scoring system which quantified the outcome of infection by this apparently uncharacteristically pathogenic clade of HRV strains.

## Results

### Genomic analyses

We undertook a comparative analysis of all HRV A2 complete coding regions (n = 6; HRV-QPM, -X1, H2, -C024, -C025 and C026) against all HRV A (n = 34), HRV B (n = 13), most HEV sequences (*n* = 71, including species A–D) and two non-human enteroviruses (simian, SEV and porcine, PEV).

HRV A2 sequences had a higher G+C content (average, 42.4%) than all other HRVs (average 38.7%) or HEV Ds and a shorter coding region (average, 2144 aa) than any other picornavirus investigated. The final residue of the HRV A2 genomes was an isoleucine, distinguishing them from all other picornavirus genomes we investigated, which ended at the preceding phenylalanine; the biological implications of this feature are unknown.

We identified 10 protease sites (data not shown) dividing the HRV A2 polyproteins into four structural and seven non-structural proteins (Figure 1A). However, unlike previous studies that relied on amino acid alignments to determine cleavage sites [33], we obtained additional evidence that these sites were recognised by 2A^pro^ and 3C^pro^ by using a neural network protease cleavage-site prediction tool. A single cleavage by 2A^pro^ was predicted between an A or L and a G residue at the VP1/2A junction. The remaining cleavages, predominantly occurring between Q and G residues, were mediated by 3C^pro^. Three motifs crucial for RNA polymerase binding (YGDD, TFLKR and SIRWT) were identified in all HRV A2 sequences [35], [36].

Using SimPlot© [37], the HRV A2 sequences were found to be most similar to those from HRV A strains (54% identity) whereas identity with HEV species ranged from 46%–49% (Figure 1B). Dips in identity occurred across all regions but only the structural region was investigated further because its components contain antigenic sites and receptor contact residues, or ‘footprints’; the latter being significant domains for defining the major and minor group HRVs [38] and other picornaviruses.

### Structural homology modelling

**Figure 2:**
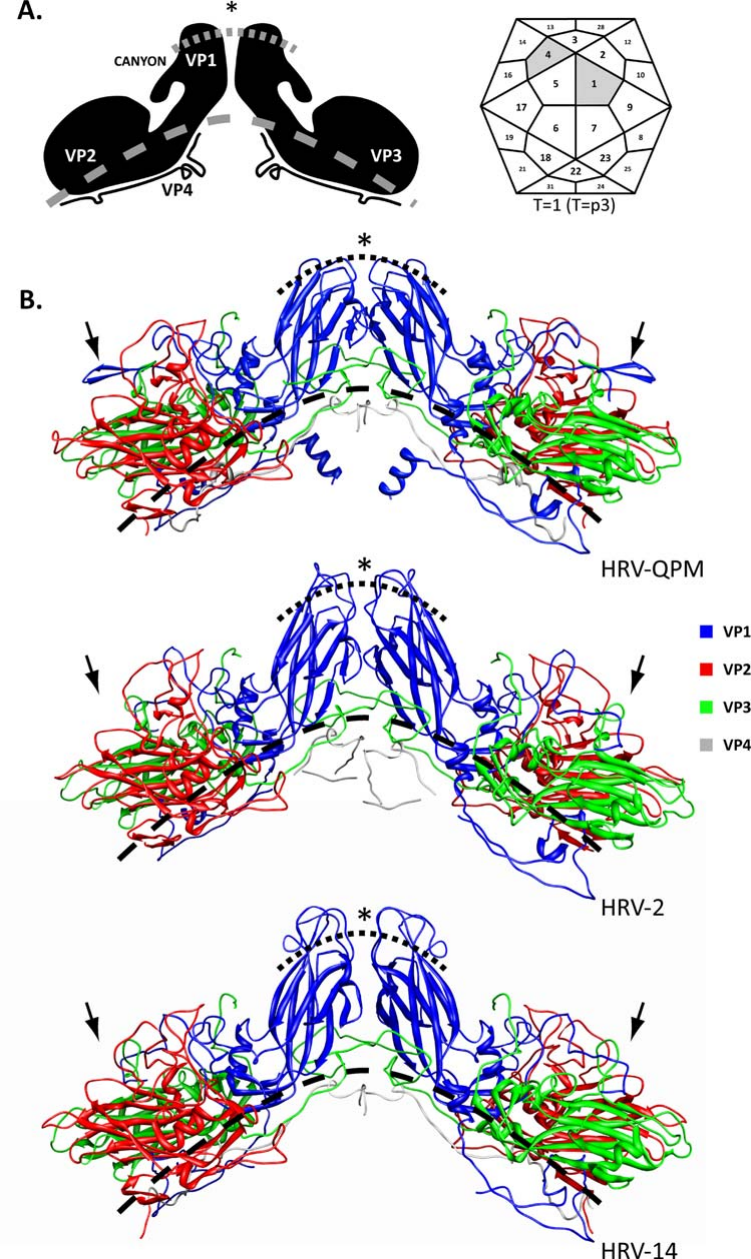
Comparison of predicted protomers. (A) A simplified depiction of two protomers in opposition on a viral capsid (shaded areas, adapted from [37]). As a guide for visualising loop length, a dashed and a dotted line are spaced equidistantly and represent proximal and distal positions from the virion core, respectively. (B) Ribbon depiction of two opposing viral protomers from HRV-QPM, HRV-2 (minor group) and HRV-14 (major group). HRV-QPM proteins were predicted by *in silico* matching to the empirically derived HRV-16 and HRV-14 structure (materials and methods). Protomers consist of one copy each of VP1, VP2, VP3 and VP4. β-sheets are depicted as flat arrows and α-helices as coiled ribbons. The formation of VP1-VP3 into eight anti-parallel β-sheets is indicative of the ‘jellyroll’ conformation typical in picornaviruses. Major differences in the predicted HRV-QPM VP1 include the shortened external loops between β-sheets (asterisk) and an additional C-terminal sheet-loop-sheet formation (arrow indicates the same location on all protomers for comparison).

**Figure 3:**
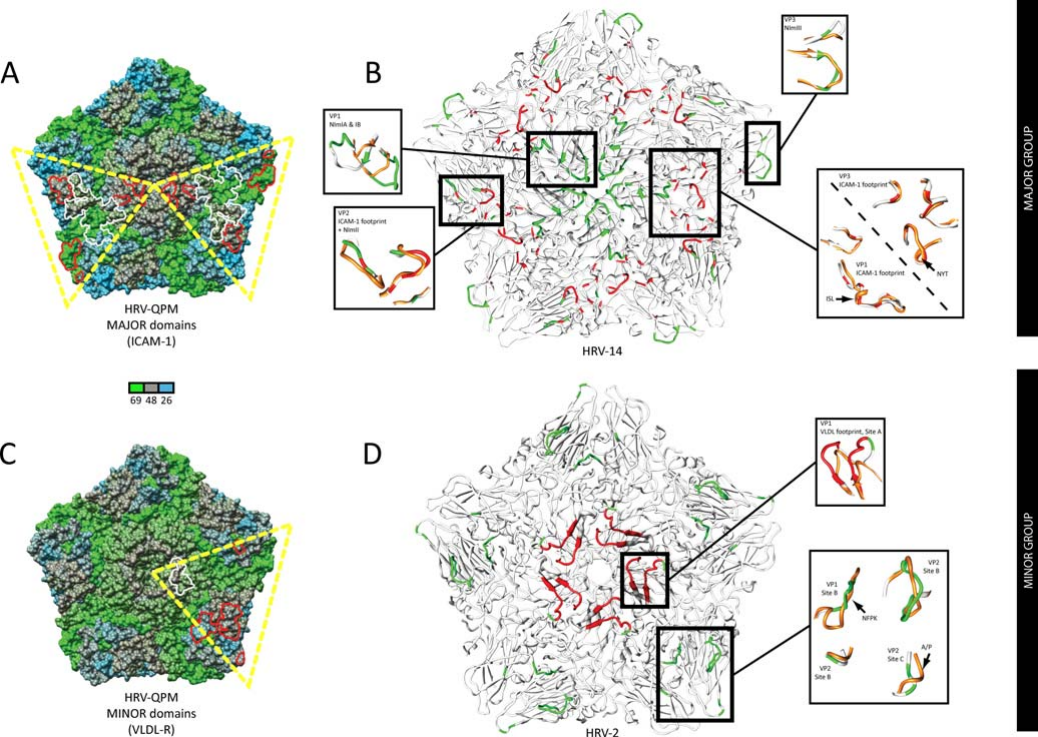
Predicted HRV-QPM pentamers compared to representative major (HRV-14) and minor (HRV-2) group HRV pentamers derived from crystallography data. (A) HRV-QPM versus HRV-14 SimPlot data projected onto a space filling depiction of the predicted HRV-QPM pentamer. Shading represents the amino acid identity (26–69%). The yellow dashed triangle represents a single icosahedral asymmetric unit (T = p3 conformation) composed of VP1 and VP2 from the same protomer and VP3 for an adjacent protomer. The major group domains of interest are divided between two asymmetric units for ease of viewing. Receptor (white) and antigenic (red) sites are shown in outline. (B) Top view ribbon depiction of a major group HRV pentamer (HRV-14; gray) with labelled antigenic neutralisation sites (NImIA-III, green) and combined HRV A (HRV-16) and B (HRV-14)ICAM-1 receptor footprints (red) [6], [37]. Magnified areas of interest (boxed) highlight computer-based predictive comparisons to the equivalent HRV-QPM (orange) structures of interest. Arrows indicate structures and corresponding sequences of interest (refer to text). (C) HRV-QPM versus HRV-2 SimPlot data projected onto the HRV-QPM pentamer. The domains of interest are mostly shown within a single asymmetric unit. (D) A minor group pentamer (HRV-2; gray) including antigenic sites (sites A–C, green) and VLDL-R footprint (red) [10]. Attachment of the VLDL-R involves adjacent VP1 molecules. Magnified VP1 area represents one half of a VLDL-R footprint [42]. Amino acid substitutions (arrowed) contributed to the differences between minor group sites B and C.

Although identifiable using amino acid alignments[33], the scope and impact of the many HRV A2 sequence deletions could not be fully qualified or quantified in this form. However, the inability to isolate the HRV A2 strains *in vitro* will hinder crystallography studies seeking to identify structural features, so we created structure-based alignments of the VP1–4 sequences from the prototype HRV A2 strain, HRV-QPM and used them to predict the presence of α-helices and β-sheets. These were identified in the eight, anti-parallel β-sheet ‘jellyroll’ conformation common among picornavirus proteins VP1, VP2 (including the EF ‘puff’) and VP3 [39] (Figure 2). Deletions in the VP1 protein imparted the most dramatic changes, reducing the protrusion of the BC, DE and HI loops compared with the other HRVs and introducing an additional C-terminal sheet-loop-sheet structure (arrow, Figure 2B). Similar deletions were noted in all HRV A2 strains but only HRV-C026 was predicted to encode a similar, C-terminal structure in VP1. We next assembled the HRV-QPM structural proteins into a protomer by associating them with the closest sequence match (HRV-16) and then replicated this structure *in silico* into a viral pentamer. RMSD values were derived from the VP1, VP2 and VP3 structures of all HRVs with crystallography-derived data available (HRV-1A, -2, -3, -14 and -16) and compared to the predicted HRV-QPM structure. HRV-QPM and HRV-16 shared the highest conformational similarity (0.145, 0.142 and 0.147 Å for the VP1, VP2 and VP3 structures respectively), reflecting the use of HRV-16 in predicting the structure HRV-QPM. The poorest agreements were between HRV-1A and HRV-3 in VP1 (0.793 Å) and VP3 (0.740 Å) and between HRV-2 and HRV-14 in VP2 (0.697 Å). At the ICAM-1 footprint, HRV-QPM values ranged from 0.064–0.106 Å and 0.709–0.779 Å in comparison to the same regions on HRV-16 and -14, respectively. Values could not be determined for HRV-2 because of the impact of deletions. We also examined the conformational agreement between an HEV footprint e.g. the CAR site employed by CV-B3, and the predicted HRV-QPM structure. A space filling model of the pentamer was next used to display SimPlot data in order to visualise regions of sequence conservation and diversification with a focus on the major (ICAM-1; Figure 3A) and minor (VLDL-R; Figure 3C) group domains. Overall, greater sequence identity was apparent between HRV-2 than HRV-14 reflecting the genomic similarity to HRV A strains, however within the domains defining HRV groups, HRV-QPM was most similar in its antigenic sites to the major group representative, HRV-14. Most sequence diversity was found in the VP2 protein while the most conserved protein in these comparisons was VP3.

To study these regions in greater detail, we mapped known HRV antigenic sites and receptor footprints onto ribbon depictions of pentamers derived from empirically determined structural data (major group, HRV-14, Figure 3B; minor group, HRV-2, Figure 3D) and compared their locations and structures to the predicted HRV-QPM pentamer. HRV-QPM and HRV-14 appeared to share a number of similar structures in the region of the ICAM-1 footprint [6], [33], [40] (Figure 3B). The noteworthy structural disparities were two small helices present in the HRV-QPM sequences; one in the VP1 and the other in the VP3 ICAM-1 footprint (Figure 3B; VP1 and VP3, ICAM-1 arrows). Similar sequences were infrequent among the other HRV strains we examined. The HRV-QPM NImIA and IB sites appeared to differ structurally due to the shortening of the BC and DE loops in VP1 while the NImII and NImIII sites appeared structurally similar.

Comparison to HRV-2 (Figure 3D) also revealed the impact of VP1 deletions. The resulting loops were shorter, which ablated any analogue of antigenic site A and may hamper binding of the VLDL-R molecule, the only LDLR family member with a well-defined footprint. The area in VP1 forming site B was altered due to the presence of more hydrophilic amino acids at the equivalent positions in HRV-QPM (Figure 3D; VP1 site B, arrow). Deletions were apparent but did not confer obvious structural changes to the VP2 portion of site B. Furthermore, in antigenic site C (VP2), a single deletion and a hydrophobic to hydrophilic amino acid change contributed to a protrusion in HRV-QPM compared to the same region in HRV-2.

### Analysis of HRV-QPM in the context of HEV receptors

Given the comparative differences we observed when locating and comparing the HRV domains, we also investigated the HRV-QPM capsid for the presence of footprints reportedly used by CV-A21 (which employs ICAM-1 as a receptor), Echovirus (EV)-11 (DAF), Poliovirus-1 (PVR) and CV-B3 (CAR).

**Figure 4:**
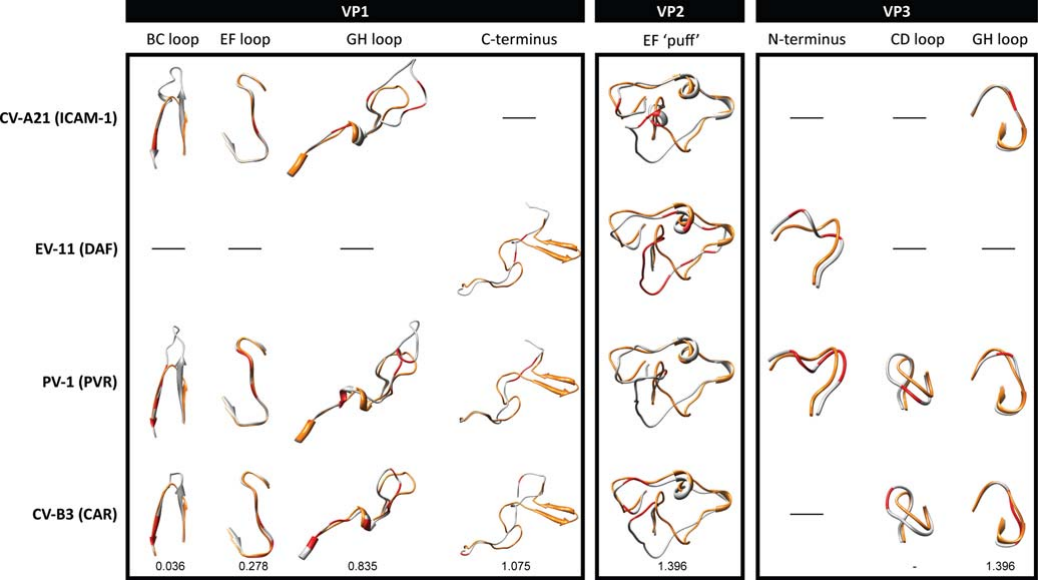
Comparison of HRV-QPM and HEV structures at the sites comprising HEV receptor footprints. Figures indicate comparison of the predicted structures of HRV-QPM (orange) with representative HEVs (gray) in regions identified as HEV receptor footprints (red) across VP1, VP2 and VP3 proteins. RMSD values shown for conformational comparison of CAR and HRV-QPM structures, in angstroms.

The deletions in the VP1 BC loop had a less obvious impact on HEV receptor footprints which were mostly located on the β-sheets rather than the loop (Figure 4). The EF loop in HRV-QPM VP1 provided the greatest observable identity with any HEV footprint while the C-terminal sheet-loop-sheet structure may interfere with binding to the HEV receptors we investigated. All the HEV VP2 receptor footprints occured in the EF ‘puff’ but in this region of HRV-QPM a number of deletions and residue substitutions were apparent (Figure 4) that affected the appearance of predicted structural overlap. HEV footprints in VP3 differed from HRV-QPM at the N-terminus and the CD loop due to amino acid substitutions and loop-shortening deletions but they overlapped in the GH loop.

### HRV-QPM capsid prediction and visualization

**Figure 5:**
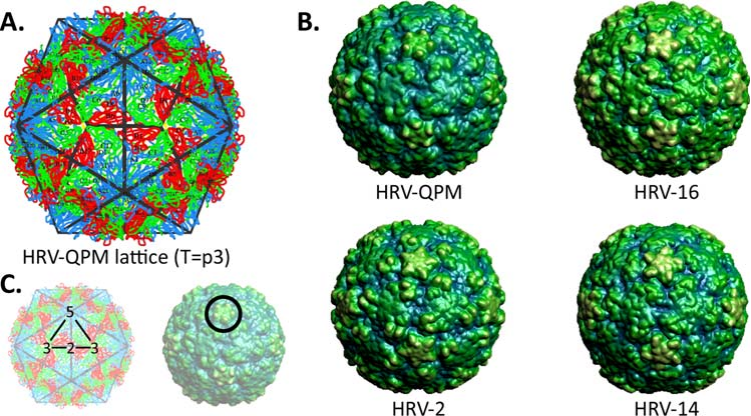
Predicted virion capsid structures. (A) Replication (×60) and mapping of the predicted HRV-QPM protomer onto a T = 1 icosahedral lattice (representing T = p3 configuration). (B) 3D rendering of predicted HRV capsids providing imagery similar to that obtained by cryo-electron microscopy reconstruction at 10 Å resolution. HRV-2, -14 and -16 predicted structures were derived from crystallography data. Each viral capsid has been depth cued to demonstrate canyon structure; yellow indicates surface detail and blue identifies areas of greatest depth. (C) Numbers superimposed over the lattice indicate the fold axis. The simplified position of the canyon is indicated by a circle on the capsid. All models were rendered and oriented identically, as determined by VIPERdb [59].

HRV-QPM protomers were replicated and mapped onto an icosahedral lattice (T = 1 configuration; Figure 5A), rendered in 3D and depth cued in order to predict capsid structures. Since HRV-QPM protein and protomer structures were inferred by homology to their closest available relatives, these capsids (HRV-16 and HRV-14) were produced from the available empirical data, using the same approach. Visual comparisons revealed that the deletions in the HRV-QPM VP1 most obviously reduced the size of protrusions around the 5-fold axis of the capsid.

### Phylogenetics

**Figure 6:**
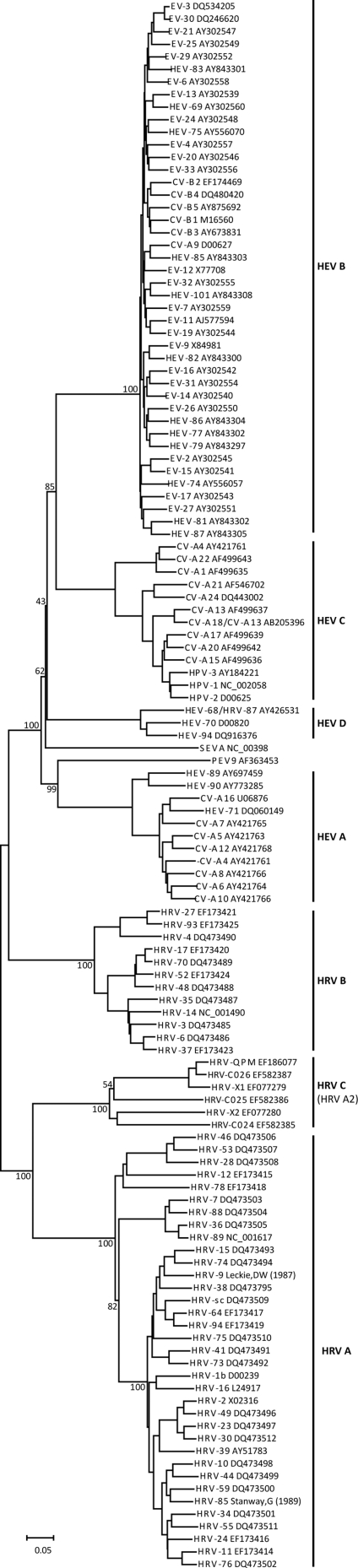
Neighbour-joining phylogeny based on representative full-length picornavirus polyprotein sequences. Trees are unrooted and relevant nodes are labelled with bootstrap values (%) (see materials and methods for details). Species are indicated next to vertical bars. CV-Coxsackievirus A; EV-Echovirus; HEV-Enterovirus; HPV-Poliovirus.

Previously, phylogenies had been estimated using subgenomic sequences from HRV A2-like strains [31]–[33]. To test whether this approach accurately represented inter-strain relationships, we compared the entire polyprotein sequences from 120 previously described and, for the first time, all six newly-identified picornavirus strains (Figure 6). We confirmed that the newly-identified HRVs occupy a distinct phylogenetic position with the genus *Rhinovirus* [34] and are most closely related to the HRV A strains (also supported by SimPlot mapping; Figure 3). We also found support for our earlier data [31] that this clade is divided into two distinct subgroups. In the 2C and 3CD regions, these subgroups share less than 70% amino acid identity, which also occurs among members of the existing HRV species.

### Clinical impact of HRV-QPM variants

Previously, preliminary and potentially subjective notes made by physicians upon first contact with ill individuals had been used for an approximate determination of the clinical impact of HRV-QPM [31]. To better address clinical impact, a comprehensive review of available patient charts was undertaken and two objective severity scoring tools were applied (Table 1). Most HRV-QPM-positive individuals (76.5%) were admitted to hospital (Table 2). Five patients were admitted for 96 hours or longer; two with severe illness and only HRV-QPM detected (variants 005 and 012) and one with moderate illness positive for HRV-QPM (variant 017) and HCoV-NL63. The remaining two had underlying medical conditions and multiple infections (HRV-QPM variant 009 and HCoV-229E with cystic fibrosis deterioration and HRV-QPM variant 008, HBoV and HCoV-NL63 with a viral URTI during admission for cardiac surgery). Oxygen therapy was required by almost half of all HRV-QPM-positive individuals studied. In addition to those described previously [31], a new co-detection was identified with the newly identified polyomavirus, WUPyV [41] in a patient exhibiting a persistent cough, already positive for HRV-QPM (variant 013) and HBoV.

**Table 1:** Criteria used to determine the severity[60] of respiratory illness upon discharge, in patients presenting to Queensland hospitals who tested positive for HRV-QPM.

Criteria	Value assigned = 0	Value assigned = 1	
Respiratory support^1^	No oxygen	Oxygen therapy	Mechanical ventilation
Intravenous fluid support^2^	No fluids	Required fluids	
Hospitalization^3^	Not admitted	Admission	≥96 hours
**Total severity score**	0–1	2–3	4–5
**Severity classification**	Mild	Moderate	Severe

1 Respiratory support by oxygen therapy and/or mechanical ventilation, ^2^Supplementary fluids delivered by either intravenous and/or nasogastric route, ^3^ [61].

**Table 2:** HRV-QPM detections and association of co-morbidities and severity of respiratory illness upon discharge.

Strain	Age(months)/Gender	Co-detection	Notes on presentation and underlying conditions	Mechanical Ventilation and treatments	Severity Score^4^
HRV-QPM001	10/M		Bronchiolitis	0^1,3^	2
HRV-QPM016	11/M	HMPV	Croup-like cough, Labs-Pert	0^1,2,3^	1
HRV-QPM015	1/M	HCoV-NL63	Croup-like cough, Labs-Pert	0^1^	2
HRV-QPM017	11/F	HCoV-NL63	Croup-like cough	0^1,2,3^	3
HRV-QPM012	5/F		Bronchiolitis, CHD	0^3^	4
HRV-QPM011	30/F		Febrile convulsion	0	1
HRV-QPM013	16/M	HBoV; WUPyV	Hacking persistent cough, Labs-Pert	0	1
HRV-QPM009	357/M	229E	CF	0	3
HRV-QPM006	7/F		Bronchiolitis, Trisomy 21, CHD, Labs-Myco	0	2
HRV-QPM003	32/M		Wheeze	0^3^	1
HRV-QPM005	815/F		Severe exacerbation of COPD	1	5
HRV-QPM004*	0/F		Bronchiolitis	0^3^	0
HRV-QPM008	13/F	HBoV; NL63	Viral URTI, CHD	1	0^5^
HRV-QPM007	55/M		Viral URTI-asthma, Labs-Myco	0^1,3^	1
HRV-QPM010	10/F		Bronchiolitis, Labs-Pert	0^1,3^	2
HRV-QPM014*	9/M		Labs-Pert	0	0
HRV-QPM002	43/M		Viral URT illness and asthma	0^1,3^	1

HMPV-Human metapneumovirus, HCoV-229E or -NL63-Human coronaviruses, HBoV-Human bocavirus, WUPyV-WU polyomavirus. Labs-Myco/Pert- laboratory testing for *Mycoplasma spp.* or *Bordetella pertussis.* ^1^Steroids, ^2^adrenalin or ^3^bronchodilators administered, ^4^Comparison of severity score using Wilcoxon rank-sum test for HRV-QPM cases with and without co-detection, p = 0.88. ^5^Recorded as ‘zero’ due to the patient being admitted for cardiac surgery and all criteria were in relation to the surgery and not the ARTI. ^*^ Not admitted. CF-cystic fibrosis. NC-No chart could be obtained for review, CHD-congenital heart disease. NN-No notes were available from presentation during the preliminary study.

### Applying species criteria to the HRV A2 strains

*In silico* data obtained from this study permitted an enhanced analysis of HRV A2 strains using the ICTV criteria which assign a member to the genus *Rhinovirus* or *Enterovirus* [42] (Table 3). The requirement common to both genera was for >70% amino acid identity in the P1 and 2C+3CD regions; this was not met by the HRV A2 genomes (Figure 1B). The remaining *Rhinovirus* species criterion was classification based on capsid-binding antiviral susceptibility and we found here, building upon earlier data [31], that all HRV A2 strains contain a Thr_191_. This residue reportedly contributes to conveying resistance to the capsid-binding antiviral Pleconaril [4], [43], placing the divergent HRVs into antiviral Group A [5].

**Table 3:** Adherence of the newly identified HRV A2s to the ICTV species demarcation criteria.

ICTV Classification	HRV A2 (***n*** ** = 6) compared to**			
**Genus ** ***Rhinovirus***	**HRV A** (*n* = 35)		**HRV B** (*n* = 12)	
>70% amino acid identity in P1	**No**		**No**	
>70% amino acid identity in 2C + 3CD	**No**		**No**	
Receptor attachment inhibited by similar antiviral agents;				
Group A	**Yes**			
Group B	**TD**			
**Genus ** ***Enterovirus***	**HEV A** (*n* = 11)	**HEV B** (*n* = 43)	**HEV C** (*n* = 13)	**HEV D** (*n* = 4)
>70% aa identity in P1	**No**	**No**	**No**	**No**
>70% aa identity in 2C + 3CD	**No**	**No**	**No**	**No**
Share a limited range of host cell receptors	**No**	**No**	**Yes**	**No**
Genome G+C composition varies ≤2.5%	**No** (5.2%)	**No** (5.5%)	**Yes** (2.3%)	**Yes** (0.5%)
Compatibility in proteolytic processing, replication, encapsidation and genetic recombination	**TD**			
Share limited range of natural hosts	**TD**			

Numbers next to species indicates the number of polyprotein sequences included in the comparison. Bracketed percentages indicate G+C content variation of the HRV A2 polyprotein coding region to reference species. TD-to be determined. ^*^ See Figure 6 for accession numbers.

Comparison to the *Enterovirus* species criteria revealed several features that may also be applicable for classifying HRV A2 strains. Firstly, although we could not identify a specific receptor using these *in silico* methods, the HRV-QPM models most closely accommodated interaction with an ICAM-1-like molecule, similar to some HEV C viruses [44]. Secondly, HRV A2 genomes exhibited ≤2.5% variation in G+C composition compared to HEV C and HEV D and ∼5% compared to HEV A and HEV B. Thirdly, HRV- and HEV-like protease cleavage sites and locations suggested similar proteolytic processing (Figure 1A). Although we have been unable to find any indication of recombination in HRV-QPM [31], the differences in amino acid identity within the novel HRVs, particularly in the 2C and 3CD region of HRV-X2, -C024 and -C025 compared to HRV-QPM, -X1 and -C026 (data not shown), may indicate a relevant event requiring further study.

In sum, these data indicate that the HRV A2 strains cannot be assigned to an existing HRV or HEV species and should be assigned to a new species, tentatively called *Human rhinovirus C*.

## Discussion

In 2007, the sequences of a number of divergent HRV strains were reported as a result of worldwide molecular investigations into cases of suspected ARTI [26], [31], [33]. Because these strains formed a distinct clade within the existing HRV As, we previously assigned to all Australian and similar New York strains, the title of HRV A2 [31]. Kistler *et al.* identified similarly divergent strains [32] and Lau et al. used sequence-based criteria to propose that some HRV A2-like strains could be classified as a new species [33]. Our ongoing efforts to characterise additional HRVs led us to catalogue the distinguishing molecular and clinical features of these HRV A2-like strains and to better address their taxonomic placement. We undertook *in sili*co analyses, for the first time using all complete HRV A2 coding sequences and performed a retrospective review of the medical records of cases from which variants of the prototype strain, HRV-QPM, had been previously detected. To date the studies reported herein are the first of their kind to have been conducted on a single HRV strain.

During our genomic analysis we found HRV A2 strains shared only 50%–53% average amino acid sequence identity with other HRV strains (Figure 1); less with any strain from an HEV species. Average HRV A2 G+C content (42.4%) fell between the rhinovirus (38.7%) and enterovirus (45.6%) genera, suggesting an intermediate evolutionary path for HRV A2 strains. Nonetheless, a large number of HRV motifs, including those recognized by the RNA-dependent RNA polymerase, were retained by HRV A2 strains. This supported the proposal that some HRV genomic regions are under purifying selection [26], even among the newly identified and divergent strains. HRV A2 strains were most similar to the HRV As overall, but amino acid sequences were as different from any traditional HRV or HEV species as the species in those genera were from each other. Previous subgenomic HRV A2 nucleotide phylogenies [31]–[33] were confirmed and expanded herein reinforcing that these NIVs reside within a distinct clade of the genus *Rhinovirus*, branching from the existing HRV A species (Figure 6).

The inability to propagate an HRV A2 virus could be due to many factors including the age, storage, site of origin and amount of the inoculum, its handling during processing, how long after symptoms appear before sampling occurs and the culture conditions used. It is also possible that an unknown primary receptor is employed or that binding of a secondary receptor is required for successful viral attachment and entry; or a mix of both. We sought to approach receptor-related issues using homology models derived crystallography data which already exist for a small number of HRV strains. Models of the component structural proteins from the prototypical HRV A2 strain, HRV-QPM, predicted that the characteristic ‘jellyroll’ conformations were retained (Figure 2) despite frequent and distinguishing deletions and inter- and intra-genus diversity at the sites of receptor contact and antigenicity (Figure 3). This also supported findings that HRV variability is mostly localized to receptor and antigenic sites [24], [33], [45] and begins to extend the data to include the more divergent strains.

The VP1 BC and HI loops receive the VLDL-R molecule among some minor group HRV strains [12], [46], [47]. The comparative reduction in size of protrusions along the 5-fold axis, resulting from deletions in the BC, DE and HI loops of the HRV-QPM VP1, together with structure-altering deletions and substitutions affecting some antigenic sites previously identified from a minor group strain (HRV-2), particularly site A, lead us to propose that HRV A2 viruses are unlikely to behave as members of the minor group. Mapping the known ICAM-1 contact sites onto the predicted HRV-QPM structure revealed that most of the footprint was retained in structure by HRV-QPM, although it differed in sequence compared to other major group strains. SimPlot data revealed that sequence variation was not uncommon among strains within the major groups. Two small α-helices predicted to form in the HRV-QPM VP1 and VP3 footprints may disrupt receptor binding but since ICAM-1 is predicted to attach at differing angles in different HRV strains [8], it may be suitably flexible to overcome such obstacles. Although NImIA and NImIB, first identified in a major group virus (HRV-14), were affected by the deletions in VP1, additional support for HRV-QPM belonging to the major HRV group came from structural similarities in the NImII (VP2) and NImIII (VP3). Amino acid substitutions occurred at these sites (E→G and G→T respectively) rather than insertions or deletions. These variations may result in distinct immunogenicity.

Localisation of four known HEV receptor footprints to the predicted HRV-QPM structure identified at least one major sequence and/or structural divergence in each (Figure 4). However, like the ICAM-1 interactions with HRV-14 and -16, HEV receptors could still bind at the same locations but utilize different amino acids on the capsid of HRV A2 strains [20].

Recent findings by Bartlett et al have shown that once past the receptor, HRV replication can occur within non-human tissues [48] exemplifying the importance of receptors in moderating the potential for more widespread HRV replication and illness. The high detection frequency of HRV A2 strains from patients with acute LRT illness may be another indication that these divergent viruses employ a tissue-specific receptor or it may be that all HRV strains cause similar illness, they simply have not been subjected to sufficient individual study. The LDLR family of molecules are located on many tissues in many species [10] and yet all these locations do not, to our knowledge, host HRV replication. Identifying an HRV A2 receptor *in vitro* cell culture system will be important for empirically addressing receptor- and replication-related issues and the outcomes may have broad implications for what is currently known of the attachment, entry and replication of HRV strains. Based on our predictive *in silico* studies of HRV-QPM, an ICAM-like molecule may be an early candidate for the role of receptor.

Future *in silico* studies could examine all available HRV polyprotein sequences to predict the extent of flexibility in receptor interactions ‘normally’ occurring in the major and minor groups. This would provide a context for the structural differences we observed in HRV-QPM. The sequence of most HRV genomes is unknown and our understanding of HRV structure is mostly due to crystallography data derived from less than 5% of HRV strains. There are limited structural data to support the wholesale extrapolation of receptor binding behaviour and capsid structure to the other HRV strains. Without data to the contrary, *in silico* analyses appear to provide a suitable surrogate to address this issue. By employing RMSD calculations to examine the conformational similarity between predicted HRV-QPM structures and those from HRV structures determined empirically, we could be confident that our predictions approximated experimental data. All comparative values (0.064–0.766 Å) were well below the maximum resolution used to originally determine HRV crystal structures (2–3 Å [14], [15], [49]). Comparison to an HEV footprint also returned values below this threshold.

While the inability to cultivate divergent HRV strains is a defining feature of the HRV A2s it is also a hindrance to classifying them. In our experience (data not shown), the earliest PCR-based methods [50] are already efficient at detecting these divergent strains. By extrapolating from published subgenomic sequence data, this clade is populated by a great number of divergent strains which are distributed both geographically and temporally, suggesting an endemic aspect to these previously uncharacterised viruses. No evidence exists to suggest that the HRV A2 strains are emerging viruses, rather it appears that they are newly identified strains that have been contributing to respiratory illness, in the absence of detection using culture-based diagnostic methods, for many years. There has now arisen a need for reliable protocols to characterise these putative viruses but in the absence of an HRV A2 isolate, neutralization, acid sensitivity and antiviral susceptibility studies, important for taxonomic placement, cannot be obtained. We have catalogued a series of features using methods which, while requiring further validation, contribute to our understanding of the biology and pathogenesis of HRV A2 strains. Additional molecular approaches will also be useful to classify other recently described divergent HRV strains [51]. Our data demonstrated that the newly identified HRVs, residing within the HRV A2 clade, do not satisfy the criteria for assignment to any existing HRV or HEV species and most likely constitute one or more novel species in the current genus *Rhinovirus*, tentatively called *Human rhinovirus C* (HRV C).

Until recently, the genus *Rhinovirus* has been frequently neglected from clinical laboratory diagnosis and to date its members are not sought independently. Improved diagnostic methods, the broader application of existing PCR technology, new sequence data and many new insights have greatly enhanced our understanding of human rhinoviruses and the selective pressures impacting on their evolution [25], [26]. Similarly, the identification of a putative new HRV species, HRV C, the characterisation of HRV-QPM's molecular features and epidemiology and the identification of similar viruses around the world [31], [34], [52] have potential to reinvigorate HRV research. We found that the variants of one strain of the HRV Cs, HRV-QPM, sought in a well characterised population of specimens collected over all months of a single year, were more often associated with LRT illness than is commonly reported. The illness frequently presented as exacerbation of expiratory wheezing or persistent cough, with a requirement for supplemental oxygen, steroids and bronchodilator treatments. Despite exhaustive PCR-based investigation for other respiratory viral causes in our previous retrospective studies [31], [41], HRV-QPM detection, in the absence of any other pathogen, occurred among infants with mild, moderate and severe (in both instances) LRT illness and most cases (88.2%) were admitted to hospital. Associated illness was usually (92.3% of the time) scored as mild to moderate and 58.8% of the HRV-QPM positive cases were children aged 12 months or less. The same age group represented 43.2% of the total study population originally screened for HRV-QPM (n = 1,244). While it is to be expected that a paediatric hospital-based population would overestimate the severity of HRV C clinical impact compared to a community-based study, our study may in fact better represent the impact of first infection with HRV strains, the major pathogens detected during the first year of life [53].

It will be important to further characterize these NIVs and determine whether their differences confer distinctive biological behaviours, such as unique growth properties, antiviral resistance and discrete clinical outcomes among infected individuals. Strain-focussed studies could also identify how many distinct HRVs circulate in a single respiratory season, how often a given strain recurs in a population and just how many HRV strains there are.

## Materials and Methods

### Nucleotide and amino acid comparisons

Nucleotide and amino acid compositions were determined using BioEdit Sequence Alignment Editor© v7.0.5.3. Picornavirus coding sequences (obtained from GenBank or picornaviridae.com) were determined by multiple alignments and subsequent truncations of the 5′and 3′UTRs. Discrepant nucleotides were substituted with the most frequently employed nucleotides, as determined by consensus alignment. Alignments available upon request. Predicted picornavirus cleavage sites were identified by amino acid sequence submission to the NetPicoRNA World Wide Web server [54].

Amino acid similarity was determined using SimPlot© v3.5 [37] employing the Hamming method on a 50% consensus of each group, a sliding window of 100 bp and a 1 bp step. Similarity data were mapped to the HRV-QPM capsid residues on the original sequence alignment and visualized using Chimera [55].

### Phylogenetic analyses

Picornavirus polyprotein sequences were compiled, translated, and aligned with the program BioEdit [56]. A neighbour-joining tree was generated using the Kimura two-parameter estimation in MEGA 3.1 [57]. Nodal confidence values (%), noted at the relevant nodes, indicate the results of bootstrap resampling (*n* = 1000). Because of the sequence diversity among the viruses analyzed, we undertook an additional analysis on a subgroup of strains whereby we gap-stripped the alignment, removing all gaps/highly divergent regions and re-estimated the phylogeny (data not shown). This confirmed the phylogenetic position determined in the first instance.

### Structural homology modelling

Secondary structures in the HRV A2 structural proteins were predicted using amino acid sequences submitted to the Jpred web server (http://www.compbio.dundee.ac.uk/∼www-jpred/submit.html). The location of α-helices and β-sheets were revealed by comparison to other picornavirus sequences using Cn3D 4.1 (http://130.14.29.110/Structure/CN3D/cn3d.shtml) (data not shown).

To determine the predicted protomer structure of HRV-QPM, DeepView/Swiss-Pdb Viewer v3.7, [58] were used to locate and individually thread HRV-QPM amino acid sequences into HRV reference structures. Of the five HRV strains with empirically determined structural data available, HRV-14 [14] and -16 [49] shared the highest sequence identities with HRV-QPM in the regions chosen. Proteins VP1 to VP3 were aligned and threaded through HRV-16 (1ayn1, 1ayn2, 1ayn3 respectively) and VP4 through HRV-14 (1na1). Models were submitted to the SWISS-MODEL server for final threading analysis. The resulting HRV-QPM protein data bank files (PDB) were then matched and structurally aligned to a template HRV protomer (HRV-16, 1AYN). Other picornavirus reference PDB files used in this study: HRV-2 (1FBN), HRV-3 (1RHI), HRV-14 (4RHV), CV-A21 (1Z7S), E-11 (1H8T), PV-1 (1HXS) and CV-B3 (1COV). Structural matching, alignments, ribbon figures, capsid predictions and root mean square deviation (RMSD) data were produced using Chimera [55].
